# The essential role of law in achieving the health-related Sustainable Development Goals

**DOI:** 10.1093/eurpub/ckaa036

**Published:** 2020-05-11

**Authors:** Dineke Zeegers Paget, David Patterson

**Affiliations:** c1 European Public Health Association—EUPHA, Utrecht, The Netherlands; c2 Global Health Law Groningen Research Centre, Faculty of Law, University of Groningen, Groningen, The Netherlands

## Abstract

In this article, we examine the essential role of law in achieving the health-related Sustainable Development Goals (SDGs). Following the World Health Organization’s broad definition of health, all SDGs can be seen to impact on human health and hence the health goal (SDG3) should be right at the centre of the entire 2030 Agenda for Sustainable Development. We note recent research on the contribution of law, including international human rights law, to achieving health for all and discuss the role of law in addressing seven emerging health challenges. Law can and should play an important role in achieving all health-related SDGs, by respecting, protecting and fulfilling the right to health, ensuring that no one is left behind.

## Health as a human right

In 2015, the United Nations’ member states adopted the 2030 Agenda for Sustainable Development, which provides a shared blueprint for peace and prosperity for all people and for the planet. Seventeen Sustainable Development Goals (SDGs) were listed for countries to react to, and act on. Law is specifically addressed in SDG 16^1^ (peace, justice and strong institutions), but it is clear that the other SDGs cannot be attained if the use and misuse of law are not addressed. In this article, we examine what law, international human rights law, can do for the health-related SDGs. We will strengthen the case of the World Health Organization (WHO) to put health at the centre of the SDGs, as health is a human right, and we will look at legal measures to ensure health for all. We also discuss the role of the law in addressing seven emerging health challenges.

The first recorded declaration of early human rights dates from 539 BC. The Cyrus Cylinder,^2^ a clay tablet, mentions the right to freedom from slavery and freedom of religion. Human rights have since developed and are now an intrinsic part of the legal structures of most societies. The 1948 Universal Declaration of Human Rights (UDHR)^3^ was established following the atrocities of World War II and is a comprehensive international affirmation of the rights to which everyone is entitled, including the right to health.

What exactly is meant by ‘the right to health’? The 1946 Constitution of the World Health Organization notes that ‘Health is a state of complete physical, mental and social well-being and not merely the absence of disease or infirmity’. The UDHR states ‘Everyone has the right to a standard of living adequate for the health and well-being of himself and his family, including food, clothing, housing and medical care and necessary social services…’ [Article 25(1)]. The right to health is thus closely related to and dependent upon the realization of other human rights, including the rights to food, housing, work, freedoms of association, assembly and movement and access health services.

There is no ‘right to be healthy’—instead the International Covenant on Economic, Social and Cultural rights (ICESCR) refers in Article 12 to ‘the right of everyone to the highest attainable standard of health’.^4^ The focus is thus on the conditions necessary for good health. These conditions are noted in multiple United Nations and regional human rights treaties. For example, housing is noted in the UDHR, the ICESCR and the Convention on the Rights of the Child. Safe and potable water is only listed in the ICESCR. Each treaty has an expert committee tasked with issuing authoritative interpretations, receiving complaints and monitoring States’ implementation of the rights in its respective treaty. Understandably, States sometimes have different interpretations of their obligations and demonstrate varying levels of commitment and implementation.

All 17 SDGs can be seen to impact on human health; however, some SDGs are directly health related. In 2019, WHO—together with 10 other international organizations—launched the Global Action Plan for Healthy Living and Well-being for All (the Plan), putting ‘the health goal (SDG 3) right at the centre of the entire 2030 Agenda for Sustainable Development’.^5^ The Plan ‘represents a historic commitment to advance collective action and accelerate progress towards the SDGs’. The European Public Health Association has also put the right to health at the forefront with a study in April 2018 tracking Europe’s progress towards the health-related SDGs, listing these as the ‘Sustainable Health Goals’.^6^ In an updated study (reference to Article no 2 in this supplement), four health issues are in need of Europe’s urgent attention: smoking, harmful use of alcohol, suicide mortality and childhood obesity.

## The role of law in ensuring health for all and leaving no one behind

Law can be used in many ways to safeguard population health and safety. Under international human rights law, countries have obligations to respect, protect and fulfil the right to health. This means that laws should not breach human rights, e.g. by instituting discrimination in access to national health services. Laws should also protect people from discrimination by third parties, e.g. the private sector, in accessing services. Finally, national laws should further countries’ international legal obligations to progressively realize the right to health, e.g. by ensuring the conditions in which people can be healthy, such as improved access to water, sanitation, housing and education. Law can also be used to regulate the environment (e.g. through air and noise pollution measures), tax unhealthy products (e.g. through fat, sugar, soda and tobacco taxes) and limit the marketing and accessibility of unhealthy products (e.g. through minimum age requirement for alcohol purchase).

Law is especially important to provide practical detail and accountability and to establish the mandate for universal health coverage (UHC).^7^Admittedly, tensions can arise between public health regulations and individual freedoms,^8^ when perceived over-regulation is sometimes referred to as the ‘Nanny State’. Nonetheless, legislation is key in fundamental questions around governance, structures and functions for operationalising the SDGs.

**Eight key messages from the 2019 Lancet–O’Neill Institute Commission on Global Health and the Law**

1. Law affects global health in multiple ways, by structuring, perpetuating and mediating the social determinants of health.
2. Although law has been central to major public health achievements in the past, its capacity to advance global health with justice remains substantially underutilized, particularly among professionals in the fields of health and science.
3. The right to health, a legally binding norm, provides a foundation for advance global health with justice and should underpin health-related legal reforms.
4. Every human being has a right to affordable, high-quality health services. By embedding equity and accountability in all health systems, the law and the rule of law can achieve health coverage that is truly universal—delivering the SDGs’ promise to leave no one behind.
5. Although the ability to enforce compliance with international legal obligations is generally limited and largely dependent on power dynamics and political will, creative mechanisms can foster compliance and help establish impetus for action.
6. Law can address the pressing health concerns of the 21st century, across diverse areas. From tobacco control, non-communicable diseases (NCDs) and road safety to health emergencies, law can implement fair, evidence-based interventions and build the research case for legal action.
7. Laws that stigmatize or discriminate against marginalized populations are especially harmful and exacerbate health disparities. The global health community must oppose laws that undermine the right to health and to equity.
8. To realize the full potential of law to advance global health with justice, the global health community should build legal capacity and establish a sustained dialogue with legislators, regulators, judges, civil society and researchers.^9^

### Twenty-first century health challenges that law needs to address

Law must adapt to address the health challenges of the 21st century.^10^ Below, we discuss seven emerging health challenges:

1. technology and digitalization,
2. deteriorating quality of life in ageing populations,
3. climate change,
4. resurgence of infectious diseases,
5. ensuring health services for hard-to-reach and vulnerable populations,
6. commercial determinants of health, and
7. threats to civil space.

#### Technology and digitalization

The speed of developing new technology and digitalization has an increasing health impact. In this section, we will focus on protection of private data and the use of genomic information for health.

In recent years, Europe has stepped up the protection of personal data. The 2018 General Data Protection Regulation^11^ aims to harmonize data privacy laws across Europe and reshape the way data is being managed. Informed consent is necessary for the use of personal data, and the right to privacy of every European citizen is hereby further strengthened. In the era of technological developments, the right to privacy is of the utmost importance.

At the same time, however, demographic research is moving towards big data, combining data from different sources to improve health analyses and thereby strengthening health interventions and planning. There is an ongoing discussion about whether informed consent to use anonymized data for a specific study also means that consent can be assumed for the use of those anonymous data for other studies (so-called ‘secondary use of data’), or whether specific consent is again necessary.

In 2015, European Public Health Association joined other non-governmental organizations (NGOs) to call for an appropriate balance that protects the interests of individuals while enabling research that benefits us all.^12^ The role of law is to create a framework to balance the right to privacy with the need for big data research (in health).

Genetic mapping is quickly developing to assist in health promotion (if you have a risk factor) and treatment (precision medicine). There is, however, a great risk that this information may lead to discrimination against individuals and population groups. For instance, Pikó et al.^13^ have reported that the Roma population has increased genetic susceptibility to venous thrombosis. Targeted interventions for Roma can be taken to consider this increased susceptibility. However, there is an increased risk of genetic discrimination, for instance by excluding venous thrombosis in health insurance for Roma.

Law should therefore regulate the use of genetic mapping and should be specifically vigilant to prevent misuse of this tool.

#### Deteriorating quality of life in ageing populations

The right to life is protected in the European Convention on Human Rights, Article 2.^14^ At the same time, the population in Europe is ageing—the average life expectancy is now 78 years. But if the increased life expectancy and the new technologies in treatment lead to the population getting older and older, should not the quality of life prevail? In other words, should the right to life include the right to die in dignity? The legal and ethical considerations of voluntary euthanasia and related advance directives should not be underestimated. Law should be vigilant to address this issue, taking into account ethical procedures and protecting against the misuse of euthanasia.

#### Climate change

The changing climate will have enormous consequences for the health of all people. Climate change must be considered in all actions to promote health. Increases in temperature will lead to increased risk of heatstroke and more tropical diseases in Europe. In 2017, the European Centre for Disease Control reported cases of malaria which were not travel-related but acquired in Europe^15^ and—with the rise in temperature—there is a risk of a further increase. Climate change will also have an impact on air pollution and emergency preparedness (e.g. flooding) and will also lead to economic migration in Europe.

Law should be used to both mitigate climate change (through regulating industry and other sources of greenhouse gases) and to ensure timely and adequate adaptation measures to protect the health of the people and of the planet.

#### Resurgence of infectious diseases

In Europe, we have seen a spectacular reduction in measles and rubella, due to childhood vaccination schemes (legal or voluntary). However, since 2017, there has been a resurgence of measles in Europe and it seems that policymakers are more influenced by fake news and anti-vax movements than by evidence. For instance, the Lorenzin law in Italy on compulsory vaccinations for children to be allowed in school, met with great controversy,^16^ but—even with a change in government—is still in place, as it protects children and whole communities against illness.

#### Ensuring health services for disadvantaged and vulnerable populations

UHC implies that everyone is able to access quality health services without experiencing financial hardship, but UHC is difficult to implement and maintain, especially in a time of budget cuts. This means that disadvantaged populations may refrain from seeking the health care they need, creating greater national health inequalities. Vulnerable communities such as migrants have even more difficulties accessing health services that accommodate language barriers and cultural differences. Finally, rural areas may face problems accessing services. The law should ensure putting a priority on financing health services and making sure that discrimination in access on any grounds is addressed.

#### Commercial determinants of health

Major NCDs in Europe include heart disease, stroke, cancer, diabetes and chronic lung disease. The rise of these NCDs has been driven by primarily four major risk factors: tobacco use, physical inactivity, the harmful use of alcohol and unhealthy diets, all linked to commercial activities of the corporate sector (the so-called ‘commercial determinants of health’).^17^

Addressing these determinants requires looking at producers, marketers and other commercial actors—rather than the individual ‘risk takers’—and formulating appropriate measures to limit commercial activities.^18^ For example, SDG target 3.4 is to ‘reduce by one-third premature mortality from NCDs…’ In the tobacco context, the Foundation for a Smoke-Free World describes itself as ‘an independent, non-profit organization committed to reducing deaths and diseases caused by smoking’ and is funded by Philip Morris International.^19^ However the Foundation is not focused on stopping tobacco use—this raises broader questions about the regulation of e-cigarettes and other tobacco products.

#### Threats to civil space

The United Nations Committee on Economic, Social and Cultural Rights, which monitors the implementation of the ICESCR, has noted (para. 54):

The formulation and implementation of national health strategies and plans of action should respect, inter alia, the principles of non-discrimination and people’s participation. In particular, the right of individuals and groups to participate in decision-making processes, which may affect their development, must be an integral component of any policy, programme or strategy developed to discharge governmental obligations under article 12…[of the ICESCR].^4^

It is well recognized that civil and political rights are part of the social determinants of health.^20^ Less well understood however is that freedoms of speech and association, guaranteed by national laws and an independent judiciary, are essential to the full achievement of the right to health. Civil society must be free to publicly challenge government action or inaction on health matters. In its watchdog role, civil society also provides a check on government inefficiency, negligence or corruption in responding to national health challenges. A free press and, today, uncensored social media, are equally vital. The rapid spread of HIV in China was in part attributed to the authorities’ censorship of early reports of unregulated blood selling.^21^^,^^22^ The experience was repeated with the SARS epidemic in 2002–2003,^23^^,^^24^ and more recently African swine fever^25^ and COVID-19^26^.

## Conclusion

As Gostin et al. state: ‘Law can be a powerful tool for advancing global health, yet it remains substantially underutilized and poorly understood’.^9^ Especially when talking about the SDGs, law has an important role to play in the field of health for all. Law is an essential tool to set a framework that allows governments and health professionals to do all they can to achieve health for all. This includes but is not limited to: protecting the rights of individuals especially the right not to face discrimination in accessing health services, assessing old and new tools to promote the public’s health and monitoring government action to make sure that no one is left behind.

It is therefore essential that health professionals and lawyers work together, understand each other and appreciate each other’s competencies. Together with an outspoken NGO community highlighting both the gaps and emerging health issues, lawyers and health professionals can together advocate for action on the health-related SDGS, making sure that no one is left behind.

*Conflicts of interest:* None declared.

## References

[1] United Nations Division for Sustainable Development Goals. Sustainable Development Goal 16. Available at: https://sustainabledevelopment.un.org/sdg16 (10 September 2019, date last accessed).

[2] Youth for Human Rights. Available at: https://www.youthforhumanrights.org/what-are-human-rights/background-of-human-rights.html (10 September 2019, date last accessed).

[3] UN General Assembly. Universal Declaration of Human Rights, 10 December 1948, 217 A (III). Available at: https://www.un.org/en/universal-declaration-human-rights (10 September 2019, date last accessed).

[4] The Committee on Economic, Social and Cultural Rights, which monitors the implementation of the ICESCR, has provided a comprehensive interpretation of Article 12. UN Committee on Economic, Social and Cultural Rights (CESCR). General Comment No. 14: The Right to the Highest Attainable Standard of Health (Art. 12 of the Covenant), 11 August 2000, E/C.12/2000/4. Available at: https://digitallibrary.un.org/record/425041?ln=en (30 December 2019, date last accessed).

[5] World Health Organization. Global Action Plan for Healthy Lives and Well-being for All (Summary). Available at: https://www.who.int/sdg/global-action-plan/GAP_summary.pdf (30 December 2019, date last accessed).

[6] European Public Health Association (EUPHA). Statement on the Sustainable Development Goals in the European Region. Available at: https://eupha.org/repository/advocacy/EUPHA_statement_on_health-related_SDGs.pdf (30 December 2019, date last accessed).

[7] Chapman AR. The contributions of human rights to Universal Health Coverage. *Health and Human Rights 2016;*18:1–15. PMC5395008 28559671

[8] Gostin LO Law and the public’s health In: Detels R, Gulliford M, Abdool Karim Q, Chuan C, editors. Oxford Textbook of Global Public Health, 6th edn. Oxford, UK: Oxford University Press, 2015: 292–302.

[9] Gostin LO, Monahan JT, Kaldor J, et al The legal determinants of health: harnessing the power of law for global health and sustainable development. Lancet 2019;393:1857–910. 31053306 10.1016/S0140-6736(19)30233-8 PMC7159296

[10] Zeegers Paget D New challenges for public health in the 21st century. In Open Access Government, July 2019. Available at: http://edition.pagesuite-professional.co.uk/html5/reader/production/default.aspx?pubname=&edid=d07278c7-9189-4e05-907f-4f904e1d685c (20 December 2019, date last accessed).

[11] European Commission. EU data protection rules. Available at: https://ec.europa.eu/info/priorities/justice-and-fundamental-rights/data-protection/2018-reform-eu-data-protection-rules/eu-data-protection-rules_en (30 December 2019, date last accessed).

[12] EUPHA. *The Times*, London, 19 November 2015. Available at: https://Eupha.org/advocacy-by-eupha (30 December 2019, date last accessed).

[13] Pikó P, Fiatal S, Kósa K, et al Genetic factors exist behind the increased risk to reduced HDL-cholesterol level in Roma population. Eur J Public Health 2016;26(Suppl 1). 10.1093/eurpub/ckw172.035 (30 December 2019, date last accessed).

[14] Council of Europe. European Convention on Human Rights. Available at: https://www.echr.coe.int/Documents/Convention_ENG.pdf (30 December 2019, date last accessed).

[15] European Centre for Disease Prevention and Control. Malaria – Annual Epidemiological Report for 2017 Available at: https://ecdc.europa.eu/en/publications-data/malaria-annual-epidemiological-report-2017 (30 December 2019, date last accessed).

[16] BBC. Italy Bans Unvaccinated Children from School, 12 March 2019 Available at: https://www.bbc.com/news/world-europe-47536981 (30 December 2019, date last accessed).

[17] Kickbusch I, Allen L, Franz C The commercial determinants of health. Lancet Glob Health 2016;4:e895–6. 27855860 10.1016/S2214-109X(16)30217-0

[18] van Schalkwyk MCI, Diethelm P, McKee M The tobacco industry is dying; disinvestment can speed its demise. Eur J Public Health 2019;29:599–600. 31348831 10.1093/eurpub/ckz006

[19] Foundation for a Smoke-Free World. Available at: https://www.smokefreeworld.org/our-vision/ (8 December 2019, date last accessed).

[20] Hahn RA, Truman BI, Williams DR Civil rights as determinants of public health and racial and ethnic health equity: health care, education, employment, and housing in the United States. SSM-Popul Health 2018;4:17–24. 29250579 10.1016/j.ssmph.2017.10.006 PMC5730086

[21] Watts J Hidden from the world, a village dies of Aids while China refuses to face a growing crisis. *The Guardian*, 25 October 2013 Available at: https://www.theguardian.com/world/2003/oct/25/aids.china (30 December 2019, date last accessed).

[22] Human Rights Watch. Restrictions on AIDS Activists in China, Vol. 17, 2005:5. Available at https://www.hrw.org/reports/2005/china0605/china0605.pdf (30 December 2019, date last accessed).

[23] Congressional-Executive Commission on China. Information Control and Self-Censorship in the PRC and the Spread of SARS. Available at: https://www.cecc.gov/publications/issue-papers/information-control-and-self-censorship-in-the-prc-and-the-spread-of-sars (30 December 2019, date last accessed).

[24] Huang Y The SARS epidemic and its aftermath in China: a political perspective In: Knobler S, Mahmoud A, Lemon S, et al, editors. Institute of Medicine (US) Forum on Microbial Threats. Learning from SARS: Preparing for the Next Disease Outbreak: Workshop Summary. Washington, DC: National Academies Press, 2004. 22553895

[25] Minter A China’s pig pandemic should worry everyone. *Bloomberg Opinion*, 24 April 2019 Available at: https://www.bloomberg.com/opinion/articles/2019-04-24/china-s-handling-of-swine-fever-outbreak-similar-to-sars (30 December 2019, date last accessed).

[26] Kavanagh MM. Authoritarianism, outbreaks, and information politics. Lancet Public Health 2020. Published online February 13, 2020. Available at: 10.1016/S2468-2667(20)30030-X (18 February 2020, date last accessed). PMC7129941 32061319

